# Altitude and risk of sudden unexpected infant death in the United States

**DOI:** 10.1038/s41598-021-81613-w

**Published:** 2021-01-25

**Authors:** Richard Johnston, Xiaohan Yan, Tatiana M. Anderson, Edwin A. Mitchell

**Affiliations:** 1 Microsoft Corporation, Redmond, WA USA; 2 Center for Integrative Brain Research, Seattle Children’s Research Institute, Seattle, WA USA; 3 Department of Paediatrics: Child and Youth Health, University of Auckland, Private Bag 92019, Auckland, 1142 New Zealand

**Keywords:** Diseases, Epidemiology

## Abstract

The effect of altitude on the risk of sudden infant death syndrome (SIDS) has been reported previously, but with conflicting findings. We aimed to examine whether the risk of sudden unexpected infant death (SUID) varies with altitude in the United States. Data from the Centers for Disease Control and Prevention (CDC)’s Cohort Linked Birth/Infant Death Data Set for births between 2005 and 2010 were examined. County of birth was used to estimate altitude. Logistic regression and Generalized Additive Model (GAM) were used, adjusting for year, mother’s race, Hispanic origin, marital status, age, education and smoking, father’s age and race, number of prenatal visits, plurality, live birth order, and infant’s sex, birthweight and gestation. There were 25,305,778 live births over the 6-year study period. The total number of deaths from SUID in this period were 23,673 (rate = 0.94/1000 live births). In the logistic regression model there was a small, but statistically significant, increased risk of SUID associated with birth at > 8000 feet compared with < 6000 feet (aOR = 1.93; 95% CI 1.00–3.71). The GAM showed a similar increased risk over 8000 feet, but this was not statistically significant. Only 9245 (0.037%) of mothers gave birth at > 8000 feet during the study period and 10 deaths (0.042%) were attributed to SUID. The number of SUID deaths at this altitude in the United States is very small (10 deaths in 6 years).

## Introduction

Despite the decline in sudden infant death syndrome (SIDS) following the Back to Sleep campaign in the early 1990s^1^, sudden unexpected infant death (SUID) remains a major cause of infant mortality in the first year of life. SUID is a broader term than SIDS. It includes three causes of death as classified by the *International Classification of Diseases*, 10th Revision (ICD-10): SIDS (R95), ill-defined causes (R99), and accidental suffocation and strangulation in bed (W75). Due to diagnostic transfer from SIDS to these other categories over the last couple of decades^2^, this definition of SUID is used by the American Academy of Pediatrics and also by the Centers for Disease Control and Prevention (CDC).

Case–control studies, particularly those in the late 1980s and early 1990s, identified several infant care practices associated with sudden infant death that were potentially modifiable. These included prone sleep position, smoking, bed sharing and the protective effect of room sharing, pacifier use and breastfeeding. Furthermore, SUID is associated with socioeconomic disadvantage and ethnic minorities.

We have previously reported that there is marked geographic variation in SUID mortality in the United States (U.S.)^3^. The variation decreased, but was not eliminated, after adjusting for covariates including known risk factors for SUID. Although there was not a clear correlation between altitude and SUID with the Rocky, Appalachian, and Sierra Nevada Mountain ranges, we recommended a deeper examination of this association in future studies. The effect of altitude on the risk of SIDS has been studied previously, but with conflicting findings. The first study from Colorado studied 268 deaths from SIDS in the postneonatal age group from 1975 through 1978^4^. The occurrence of SIDS was not influenced by whether the infant was born, resided, or died at altitudes greater than 7500 feet. In contrast, a study from Nebraska of 132 SIDS cases between 1973 and 1978 found that the incidence of SIDS increased with increasing altitude^5^. An Austrian study of 99 SIDS cases between 1984 and 1994 showed that SIDS mortality increased with altitude for infants sleeping prone, but not for those sleeping non-prone^6^. A more recent study from Colorado found a two-fold increased risk of SIDS for infants of mothers that are resident at greater than 8000 feet^7^. However, the reported increased risk at > 8000 feet was based on just six deaths certified as SIDS. There was no statistically significant increased risk of SUID with high altitude.

The aim of this study was to examine whether the risk of SUID varies with altitude in the U.S. We hypothesised that increasing altitude would be associated with an increased risk of SUID.

## Methods

Data from the CDC’s Cohort Linked Birth/Infant Death Dataset^8^ and the National Center for Health Statistics for births between 2005 and 2010 were examined^9^. This dataset links information from birth certificates on all live births with information from death certificates on all infant deaths occurring in the U.S. SUID was defined as deaths from SIDS (R95), ill-defined causes (R99) or accidental suffocation and strangulation in bed (W75). SUID in the early neonatal age group (< 7 days) were excluded, as the epidemiology of these cases differ from those who died at ≥ 7 days^10^. Deaths over one year of age were also excluded.

Controls were infants surviving through to the first birthday.

Adjusted odds ratios (aOR) were calculated using logistic regression and generalized additive model (GAM) for the whole of the U.S., adjusting for mother’s race, Hispanic origin, marital status, age, education and smoking, father’s age and race, number of prenatal visits, plurality, live birth order, and infant’s sex, birthweight and gestation and year. We used the mgcv package^11^ in R^12^ to perform the model fitting and analysis for GAM with a binomial family and a thin plate splines smoother for altitude:

$$logit\left(SUID\right)=intercept+smoother\left(altitude\right)+other \;variables$$

County of birth was used to estimate altitude at the county-level^13^. Altitude was categorised by < 6000 feet, 6000–8000 feet and > 8000 feet. The GAM analysis used the same altitude variable, but coded as a continuous numerical variable via a smoothing function rather than categorised. The smoothing function allowed us to obtain a continuous estimation of the altitude effect over SUID.

All data used were deidentified and in the public domain, and thus ethical approval was not required.

## Results

In the study period (6 years) there were 25,305,778 live births and 23,673 deaths (rate = 0.94/1000 live births). In total, 9245 (0.037%) of mothers gave birth at > 8000 feet. Over these 6 years there were only 10 SUID cases that occurred over 8000 feet. There was a small, but statistically significant, increased risk of SUID with altitude > 8000 feet compared with < 6000 feet (aOR = 1.93; 95% confidence interval (CI) 1.00–3.71). Infants born at an elevation of 6000 to 8000 feet were not at increased risk of SUID (aOR = 0.97: 95% CI 0.83–1.12). Supplementary Table 1 provides the aORs for the covariates in the logistic model. The altitude of counties in the U.S. is shown graphically in Supplementary Figure 1.

**Figure 1:**
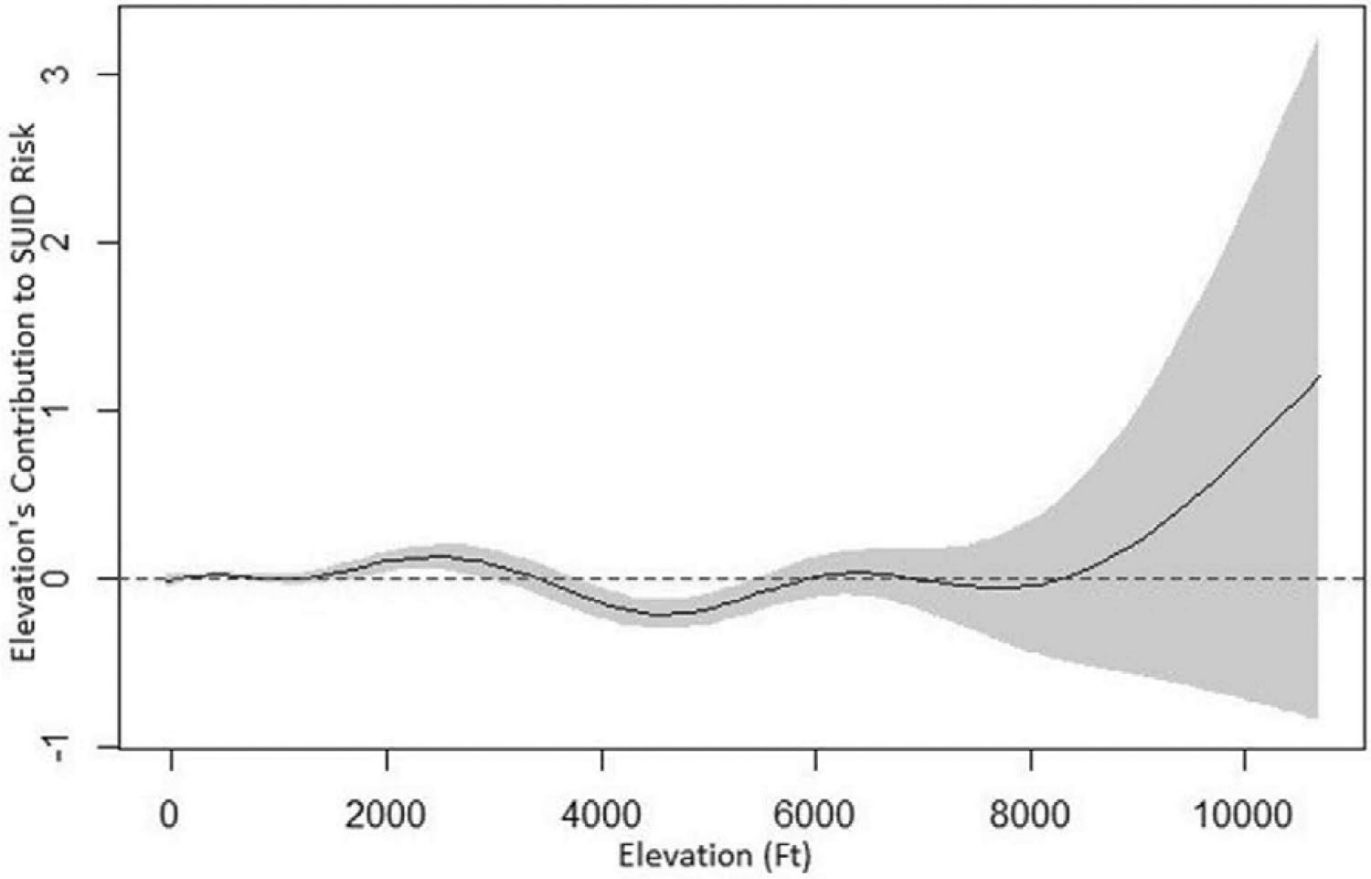
Generalized additive model showing the change in risk of SUID with altitude (solid line with 95% confidence interval). The dashed line is the average risk for the U.S. over the study period (2005–2010).

The GAM for the U.S. showed a statistically significant smoothing term over elevation. The smoothing term allows a more flexible functional form over elevation in explaining SUID risk, after accounting all other covariates. To visualize the contribution of altitude to SUID, we plotted the estimated smoothing function value of altitude in Fig. 1 along with 95% confidence bands. A negative smoother value for altitude corresponds to lower than national average SUID risk, whereas the positive smoother value indicates higher than average risk. Figure 1 shows a statistically significant increased risk of SUID between 1800 and 2900 feet, and lower risk between 3800 and 5400 feet (altitude has been rounded to the nearest 100 feet). As in the logistic model there is an increased risk over 8000 feet, but this was not statistically significant.

## Discussion

This analysis supported our hypothesis that there would be an increased risk of SUID at higher altitude, however, the effect was small and only seen at > 8000 feet in the logistic regression model. It should be noted that few (0.037%) babies were born at high altitudes, and that very few SUID cases occurred at these high altitudes (10 in 6 years in the U.S.).

The GAM model also showed a non-statistically significant increased risk over 8000 feet. Unexpectedly, we observed a significantly increased risk of SUID with maternal residence between 1800 and 2900 feet, and lower risk between 3800 and 5400 feet. Although statistically significant, the magnitude in the change in risk is very small. We cannot explain this finding, and suspect it may be a chance finding in part due to the large sample size.

An increased risk of SUID with high altitude has biological plausibility. It might be due to maternal hypoxia in pregnancy affecting the fetus, such as a lowering birthweight^14,15^. Alternative mechanisms might include reduced oxygen availability in the infant^16^ or exposure to a colder environmental temperature^17^.

Fetal haemoglobin (HbF) is the main oxygen carrier in the fetus, and levels remain high until about 2–4 months after birth^18^. It has a higher oxygen affinity than adult haemoglobin (HbA). While some studies have found no difference in HbF concentration between SIDS cases and controls^19–21^, several others have found increased concentrations of HbF in tissues from SIDS infants postmortem^22–24^. HbF levels have been shown to be higher in infants with chronic hypoxemia^22^ and adults living at high altitude^25^. We speculate that infants living at higher altitudes have increased concentrations of HbF and thus their blood has a greater affinity for oxygen and may not release oxygen to the tissues as readily as blood composed almost exclusively of HbA. This potentially could lead to an increased vulnerability to hypoxia.

This study is the largest study that has examined the effect of altitude on the risk of SUID, and encompassed the whole of the U.S. It included over 25 million births and 23,673 SUID cases. Furthermore, the study was able to adjust for a number of established risk factors for SUID. The major limitation was that we used a proxy measure for altitude, namely the altitude at county level of the location of the baby’s birth. Altitude at the location the infant died might be more relevant. A further limitation was that we were unable to adjust for factors related to infant care, such as sleep position, bed sharing and pacifier use.

## Conclusions

Given that so few cases occur at these high altitudes in the U.S., future research and educational resources should focus on SUID risk factors with higher prevalence. However, the findings may be relevant to other countries with larger populations residing at high altitude.

## Supplementary Information

Supplementary Figure S1.

Supplementary Table S1.

## Abbreviations

CDC: Centers for Disease Control and Prevention
CI: Confidence interval
GAM: Generalized additive model
ICD-10: *International Classification of Diseases*, 10th Revision
aOR: Adjusted odds ratio
SIDS: Sudden infant death syndrome
SUID: Sudden unexpected infant death
U.S.: United States
