# Supplementary material for: Altitude and risk of sudden unexpected infant death in the United States
Source: Sci Rep. 2021 Jan 25;11:2161. doi: 10.1038/s41598-021-81613-w (PMC7835371; doi:10.1038/s41598-021-81613-w)

Supplementary Fig 1. Geographic variation of altitude by county in the U.S. Dark blue represents altitude <6,000 feet, medium blue 6,000-8,000 feet and light blue >8,000 feet. There are no data for the counties colored grey. Software: R 3.5.0 (r-project.org). Package: usmap (cran.r-project.org/web/packages/usmap/)

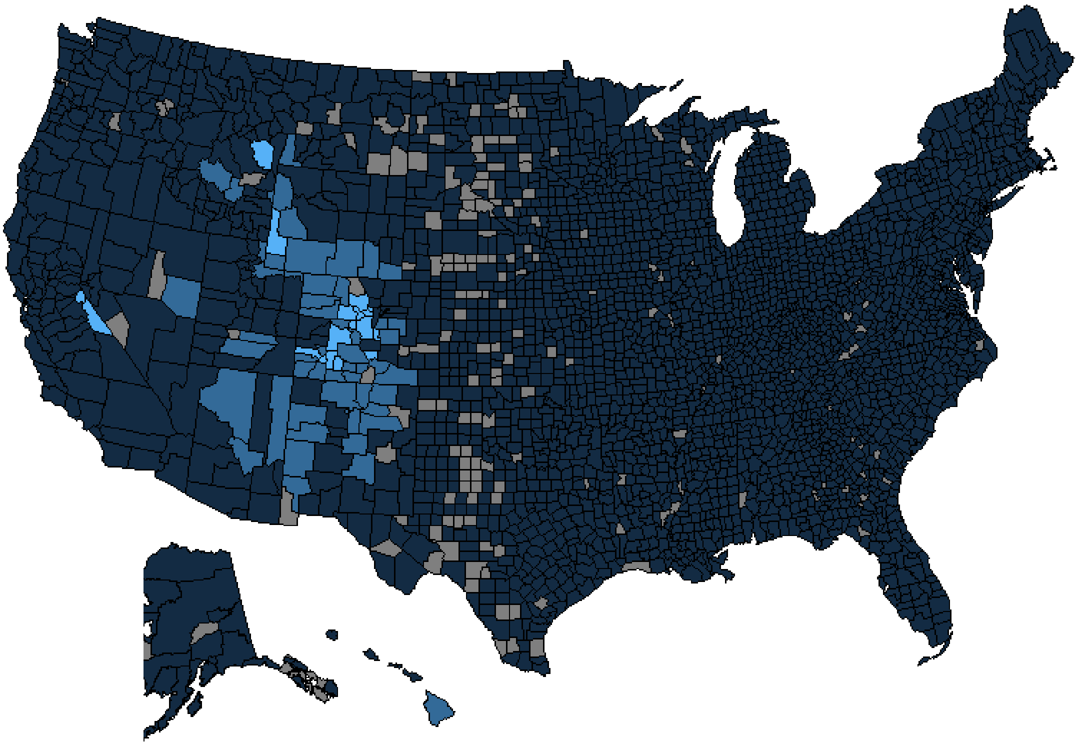

Supplement: Supplementary file 1 — Supplementary Figure S1. [file 41598_2021_81613_MOESM1_ESM.pdf]
