# Supplementary material for: Altitude and risk of sudden unexpected infant death in the United States
Source: Sci Rep. 2021 Jan 25;11:2161. doi: 10.1038/s41598-021-81613-w (PMC7835371; doi:10.1038/s41598-021-81613-w)
Supplement: Supplementary file 2 — Supplementary Table S1. [file 41598_2021_81613_MOESM2_ESM.pdf]

Supplementary Table 1. Adjusted ORs and 95% CI for the covariates in the logistic model.

	aOR	95% CI	p value
**Elevation**			
Greater than 8000 feet	1.93	1.00-3.71	0.05
6000 - 8000 feet	0.97	0.83-1.12	0.65
Lower than 6000 feet	Ref		
**Year**			
2005	0.97	0.93-1.02	0.21
2006	0.96	0.92-1.01	0.1
2007	Ref		
2008	1.02	0.98-1.06	0.41
2009	0.98	0.93-1.02	0.32
2010	0.96	0.91-1.00	0.06
**Father's Age (years)**			
<15	1.43	0.53-3.83	0.48
15-19	1.48	1.36-1.59	<0.001
20-24	1.37	1.30-1.44	<0.001
25-29	1.17	1.11-1.23	<0.001
30-34	Ref		
35-39	0.96	0.90-1.03	0.27
40-44	1.07	0.97-1.17	0.17
45-49	1.04	0.90-1.19	0.63
50-54	1.24	0.99-1.55	0.06
55-98	1.35	0.96-1.90	0.08
Not stated	1.75	1.62-1.88	<0.001
**Birthweight (g)**			
1499 or less	2.41	2.17-2.68	<0.001
1500-2499	1.92	1.84-2.00	<0.001
2500 or more	Ref		
Unknown or not stated	2.84	1.53-5.24	<0.001
**Smoked during pregnancy**			
Not on certificate	1.15	1.04-1.27	<0.05
Unknown or not stated	1.06	1.01-1.12	<0.05
No	Ref		
Yes	2.13	2.04-2.22	<0.001
**Mother's Age (years)**			
10-12	7.14	2.66-19.16	<0.001
13	2.49	1.24-5.00	<0.05
14	3.55	2.71-4.65	<0.001
15	3.06	2.59-3.61	<0.001
16	2.69	2.38-3.06	<0.001
17	2.63	2.37-2.92	<0.001
18	2.80	2.56-3.07	<0.001
19	2.37	2.18-2.58	<0.001

20	2.28	2.10-2.47	<0.001
21	2.13	1.97-2.31	<0.001
22	1.82	1.68-1.98	<0.001
23	1.69	1.56-1.83	<0.001
24	1.46	1.34-1.58	<0.001
25	1.32	1.22-1.44	<0.001
26	1.18	1.09-1.29	<0.001
27	1.09	1.00-1.18	0.06
28	Ref		
29	0.89	0.81-0.98	<0.05
30	0.79	0.72-0.87	<0.001
31	0.76	0.68-0.84	<0.001
32	0.64	0.57-0.72	<0.001
33	0.66	0.59-0.74	<0.001
34	0.56	0.49-0.63	<0.001
35	0.54	0.48-0.62	<0.001
36	0.59	0.51-0.67	<0.001
37	0.53	0.46-0.62	<0.001
38	0.54	0.46-0.64	<0.001
39	0.52	0.43-0.63	<0.001
40	0.48	0.39-0.60	<0.001
41	0.40	0.30-0.52	<0.001
42	0.43	0.31-0.59	<0.001
43	0.32	0.20-0.50	<0.001
44	0.33	0.18-0.61	<0.001
45	0.22	0.08-0.60	<0.001
46	0.00	0-1.276E+45	0.86
47	0.40	0.10-1.59	0.19
48	0.37	0.05-2.61	0.32
49	0.60	0.08-4.25	0.61
50	0.00	0-4.963E+86	0.92
**Gestation (weeks)**			
17	0.30	0.08-1.22	0.09
18	0.10	0.01-0.71	<0.05
19	0.20	0.06-0.63	<0.05
20	0.24	0.11-0.54	<0.001
21	0.22	0.10-0.46	<0.001
22	0.22	0.11-0.43	<0.001
23	0.42	0.27-0.65	<0.001
24	1.08	0.84-1.39	0.53
25	1.00	0.78-1.29	0.97
26	0.88	0.69-1.13	0.32
27	1.05	0.85-1.31	0.64
28	1.05	0.86-1.28	0.64
29	1.39	1.18-1.63	<0.001
30	1.22	1.05-1.42	<0.05

31	1.35	1.19-1.54	<0.001
32	1.33	1.19-1.49	<0.001
33	1.31	1.18-1.44	<0.001
34	1.28	1.18-1.39	<0.001
35	1.31	1.22-1.41	<0.001
36	1.36	1.28-1.44	<0.001
37	1.24	1.18-1.30	<0.001
38	1.11	1.07-1.16	<0.001
39	Ref		
40	0.93	0.89-0.98	<0.001
41	1.00	0.95-1.06	0.95
42	1.05	0.96-1.14	0.3
43	1.18	1.06-1.31	<0.001
44	1.06	0.91-1.23	0.44
45	1.09	0.89-1.33	0.41
46	1.16	0.89-1.51	0.27
47	1.30	0.95-1.79	0.1
Unknown	1.03	0.82-1.30	0.78
**Mother's Education**			
8th grade or less	0.79	0.69-0.90	<0.001
9th-12th grade, no diploma	1.05	0.95-1.17	0.31
High school graduate or GED completed	0.99	0.90-1.10	0.9
Some college credit, no degree	0.95	0.85-1.05	0.32
Associate degree	0.87	0.76-0.99	<0.05
Bachelor's degree	0.69	0.61-0.79	<0.001
Master's degree	0.59	0.49-0.71	<0.001
Doctorate or professional degree	0.53	0.37-0.74	<0.001
Unknown	0.88	0.72-1.07	0.21
Not on certificate	Ref		
**Plurality**			
Single	Ref		
Twin	1.05	0.99-1.12	0.1
Triplet	0.78	0.56-1.07	0.12
Quadruplet	0.33	0.05-2.38	0.27
Quintuplet or higher	0.00	0-1.397E+235	0.97
**Marital Status**			
Married	Ref		
Unmarried	1.41	1.36-1.46	<0.001
**Father's Race**			
White	Ref		
Black	1.34	1.28-1.41	<0.001
American Indian or Alaskan Native	1.25	1.11-1.41	<0.001
Asian or Pacific Islander	0.85	0.75-0.97	<0.05
Unknown or Unstated	1.02	0.96-1.08	0.59
**Mother's Race**			
White	Ref		

Black	0.94	0.91-0.98	<0.05
American Indian or Alaskan Native	1.18	1.07-1.30	<0.001
Asian or Pacific Islander	0.83	0.74-0.92	<0.001
**Live Birth Order**			
1	Ref		
2	2.02	1.95-2.10	<0.001
3	2.84	2.73-2.97	<0.001
4	3.73	3.54-3.93	<0.001
5	4.36	4.06-4.69	<0.001
6	4.94	4.46-5.47	<0.001
7	5.24	4.52-6.07	<0.001
8 or more	5.96	5.13-6.93	<0.001
Unknown	1.85	1.57-2.18	<0.001
**Number of Prenatal Visits**			
None	1.53	1.42-1.65	<0.001
1-2	1.44	1.32-1.57	<0.001
3-4	1.42	1.33-1.52	<0.001
5-6	1.35	1.28-1.43	<0.001
7-8	1.19	1.13-1.25	<0.001
9-10	1.09	1.04-1.13	<0.001
11-12	Ref		
13-14	0.96	0.91-1.00	0.07
15-16	1.02	0.96-1.08	0.48
17-18	0.99	0.89-1.10	0.82
19+	1.13	1.04-1.23	<0.001
Unknown or Not Stated	1.18	1.10-1.26	<0.001
**Infant Sex**			
Female	0.72	0.71-0.74	<0.001
Male	Ref		
**Mother's Hispanic Origin**			
Non-Hispanic	Ref		
Mexican	0.42	0.40-0.44	<0.001
Puerto Rican	0.65	0.59-0.72	<0.001
Cuban	0.66	0.50-0.87	<0.001
Central or South American	0.34	0.30-0.38	<0.001
Origin Unknown or Not Stated	0.68	0.62-0.74	<0.001
Other and Unknown Hispanic	0.91	0.78-1.06	0.23
